# ﻿Incongruent molecular and morphological variation in the crab spider *Synemaglobosum* (Araneae, Thomisidae) in Europe

**DOI:** 10.3897/zookeys.1078.64116

**Published:** 2021-12-17

**Authors:** Karin Urfer, Tamara Spasojevic, Seraina Klopfstein, Hannes Baur, Liana Lasut, Christian Kropf

**Affiliations:** 1 Natural History Museum Bern, Bernastrasse 15, 3005 Bern, Switzerland Natural History Museum Bern Bern Switzerland; 2 University of Bern, Institute of Ecology and Evolution, Baltzerstrasse 6, 3012 Bern, Switzerland University of Bern Bern Switzerland; 3 Natural History Museum St.Gallen, Rorschacher Strasse 263, 9016 St.Gallen, Switzerland Natural History Museum Basel Basel Switzerland; 4 Natural History Museum Basel, Augustinergasse 2, 4051 Basel, Switzerland Natural History Museum St.Gallen St.Gallen Switzerland

**Keywords:** DNA barcoding, genital organs, MRA, multivariate ratio analysis, PCA, principal component analysis, species delimitation, spider taxonomy

## Abstract

Establishing species boundaries is one of the challenges taxonomists around the world have been tackling for centuries. The relation between intraspecific and interspecific variability is still under discussion and in many taxa it remains understudied. Here the hypothesis of single versus multiple species of the crab spider *Synemaglobosum* (Fabricius) is tested. The wide distribution range as well as its high morphological variability makes this species an interesting candidate for re-evaluation using an integrative approach. This study combines information from barcoding, phylogenetic reconstruction based on mitochondrial CO1 and ITS2 of more than 60 specimens collected over a wide range of European localities, and morphology. The findings show deep clades with up to 6% mean pairwise distance in the CO1 barcode without any biogeographical pattern. The nuclear ITS2 gene did not support the CO1 clades. Morphological assessment of somatic and genital characters in males and females and a morphometric analysis of the male palp uncovered high intraspecific variation that does not match the CO1 or ITS2 phylogenies or biogeography either. Screening for endosymbiotic *Wolbachia* bacteria was conducted and only a single infected specimen was found. Several scenarios might explain these inconsistent patterns. While the deep divergences in the barcoding marker might suggest cryptic or ongoing speciation or geographical isolation in the past, the lack of congruent variation in the nuclear ITS2 gene or the studied morphological character systems, especially the male palp, indicates that *S.globosum* might simply be highly polymorphic both in terms of its mtDNA and morphology. Therefore, more data on ecology and behaviour and full genome sequences are necessary to ultimately resolve this taxonomically intriguing case.

## ﻿Introduction

To assess species richness is an urgent duty to manage and conserve biodiversity. Estimation of species richness using various extrapolation methods is one way to tackle the issue. One influential work of this kind was carried out by Erwin (1982), who estimated from canopy fogging in Panama that the number of extant arthropod taxa may be as high as 30 million. The only way to verify such estimates is to approach real numbers by describing new species. However, assessing species numbers is often difficult, especially in the case of hyper-diverse groups like arthropods (Colwell 1994).

Traditional taxonomy, which is mainly based on morphological traits, was the most effective way to describe new species before molecular techniques became widely applicable. Nowadays, combining these two approaches has become the most powerful method taxonomists use to identify, delimitate, and describe new species (Dayrat 2005; Schlick-Steiner et al. 2010; Gokhman 2018). However, there are numerous ways in which morphological and molecular data can disagree which poses challenges to taxonomists (Funk and Omland 2003; Hebert et al. 2003; Fleck et al. 2006). This became especially clear with the advent of DNA barcoding in the last years (Blagoev et al. 2009).

DNA barcoding is nowadays a very common method for species identification, based on the analysis of a short genetic fragment (Coddington 1996; Hebert et al. 2003; Čandek et al. 2013). Usually, this fragment is the cytochrome c oxidase 1 gene (CO1) located in the mitochondrial DNA (mtDNA). To identify a specimen, the sequenced barcoding fragment is compared with an existing library. There were intensive sampling and sequencing efforts to build a universal barcode library, i.e., Barcode of Life Data Systems (BOLD) (Ratnasingham and Hebert 2007, 2013; Blagoev et al. 2016), that provides a cheap tool to identify specimens quickly with a single-locus approach. Beside this, DNA barcoding can also aid species discovery (Dayrat 2005; Tyagi et al. 2019), but the suitability of this method in closely related species or within a species complex is still under discussion (Whitworth et al. 2007; Slowik and Sikes 2015; Spasojevic et al. 2016; Gibbs 2018). Potential obstacles for barcoding as the sole tool for species discovery include incomplete lineage sorting (Galtier et al. 2009), recent species divergence in big population size organisms (Maddison 1997), and endosymbiont-mediated introgressive hybridisation (Hurst and Jiggins 2005; Goodacre et al. 2006; Klopfstein et al. 2016). Another constraint is that barcodes sometimes reflect biogeographical patterns instead of species specificity (Nicholls et al. 2012; Collins and Cruickshank 2013).

There are two major ways in which barcoding, and morphology can disagree (Funk and Omland 2003; Hogner et al. 2012). The first scenario concerns morphologically clearly separable species, but the interspecific variability of the barcode is low or even zero. The second scenario includes morphologically cryptic species that can only be identified with molecular data, or with additional data that demonstrate mating barriers between the species (e.g., behavioural data or chemical volatiles) (Töpfer-Hofmann et al. 2000; Kunz et al. 2012). Both patterns have been reported in a diverse array of organisms, including spiders (Slowik and Sikes 2015; Ivanov et al. 2018). When providing equivocal results, barcoding studies should be evaluated carefully, i.e., by multilocus approaches or by analysis of morphological variation (Lefébure et al. 2006; Dellicour and Flot 2018).

With almost 50'000 described species (World Spider Catalog 2021), spiders are the second largest group of arachnids after mites. From the mid of the 18^th^ century (Clerck et al. 1757) until today, their copulatory organs have been successfully used for species delimitation and description. Unfortunately, that means that juveniles are mostly impossible to identify, and females may also cause problems in certain groups. Therefore, the barcoding approach is especially popular among arachnologists (Hebert et al. 2003; Blagoev et al. 2016).

The crab spider *Synemaglobosum* (Fabricius, 1775) shows a Palaearctic distribution, ranging from Western Europe to Eastern Asia (Ono 1988, World Spider Catalog 2019). Juvenile individuals show some ballooning behaviour (Blandenier and Fürst 1998) that can explain the wide distribution range. Next to the wide distribution, *S.globosum* shows high polymorphism in colour pattern and in the morphology of copulatory organs; this is reflected in many synonyms and subspecies names (World Spider Catalog 2021). Integrative taxonomy could help in structuring this variation and may even lead to the discovery of previously overlooked, cryptic species.

Here, we present the results of a combined molecular (based on the two markers CO1 and ITS2), morphological and morphometrical study on the variation in *S.globosum* over a wide range of sampling localities. Additionally, we perform a screening for the bacterial endosymbiont *Wolbachia* to examine its potential influence on intraspecific variation in the mitochondrial CO1 gene.

## ﻿Methods

### ﻿Data collection

**Figure 1.**
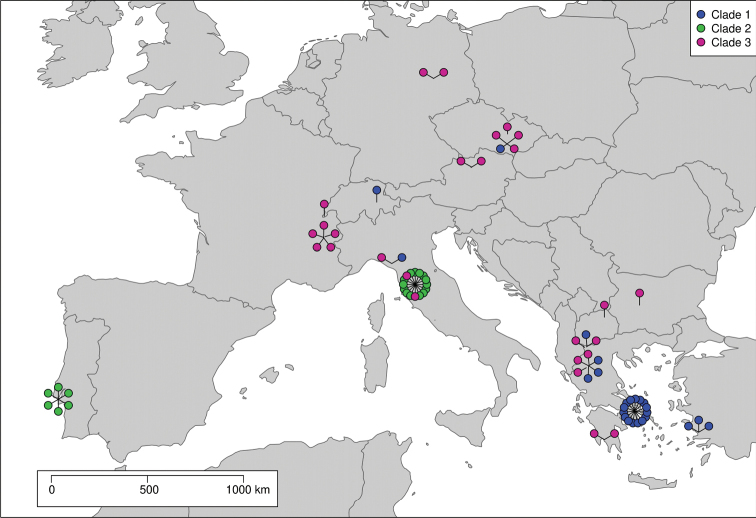
Map of localities of 72 Synemaglobosum individuals used for the CO1 phylogeny. The specimens were collected in Portugal, France, Italy, Czech Republic, North Macedonia, Greece, and Turkey. Sequences of specimens from Switzerland, Austria, Germany, and Bulgaria were obtained from BOLD. The colours correspond to the three clades in the CO1 phylogeny of S.globosum.

Seventy-two adult *S.globosum* individuals were collected across the species range within Europe, including Portugal, France, Italy, Cech Republic, North Macedonia, Turkey, and Greece (Fig. 1; Suppl. material 1). Portuguese specimens were provided from the University of Nottingham. The distance between the two most distant sample sites was 3'100 km. Calculation of geographical distance was done with QGIS (QGIS Development Team, 2018) and the map shown in Fig. 1 was created in R version 4.0.3, using the packages maps v. 3.3.0 and sp v 1.4.5 (Pebesma et al. 2005; Becker et al. 2018; RCore team 2020). After capture, the specimens were preserved in 100% ethanol and stored at -80 °C. Specimens were identified using Levy (1985), Utochkin (1960b), Mcheidze (1997) accessed via World Spider Catalog 2021 and araneae, Version 02.2021 (Nentwig et al. 2021). From each spider, two legs were used for DNA extraction. The extracted DNA was then stored at -20 °C at the Natural History Museum of Bern, Switzerland.

### ﻿Laboratory protocols

For DNA extraction, 180 µl buffer and 20 µl protease K according to Qiagen Easy Cube digestion protocol were used to digest the two legs. Digestion duration was 14–16 h at 56 °C. After digestion, DNA was purified with the Qiagen Easy Cube following the rodent blood and tissue protocol.

The PCR mixture was composed as follows: 12.5 µl GoTaq Hot Start Green Master Mix (Promega, Switzerland), 6.5 µl nuclease free water, 2 µl DNA and 2 µl forward and reverse primer (10 µM). The PCR conditions were an initial denaturation for two minutes at 94 °C, 35 cycles with a denaturation phase for 30 sec at 94 °C, an annealing phase for 30 seconds at adequate temperature for each primer (Table 1), and an elongation phase for 45 seconds at 72 °C. For the CO1 primer pair LCO1490/Chelicerata2R, five pre-cycles with the higher annealing temperature were included. The final elongation cycle was for 10 minutes at 72 °C. The quality of the PCR product was tested on a 1% agarose gel where 1.6 µl of the dye Midori Green (Nippon Genetics, Europe) was added. The obtained PCR products were sequenced in both directions by LGC Genomics in Berlin, Germany.

**Table 1.** Primer sequences with references and annealing temperatures.

Gene Primer	Forward (F) Reverse (R)	Sequence 5’–3’	Reference	Annealing °C
**CO1**	–	–	–	–
LCO1490	F	GGTCAACAAATCATAAAGATATTGG	(Folmer et al. 1994)	50/48 °C
ChelicerataR2	R	GGATGGCCAAAAAATCAAAATAAATG	(Barrett and Hebert 2005)	–
C1-J- 2183	F	CAACATTTATTTTGATTTTTTGG	(Folmer et al. 1994)	47 °C
C1-N-2778	R	GGATAATCAGAATATCGTCGAGG	(Simon et al. 1994), (Barrett and Hebert 2005)	–
**ITS2**	–	–	–	–
ITSf	F	TCCTCCGCT TATTTATATGC	(Agnarsson 2010)	50 °C
ITSr	R	GGGTCGATGAAGAACGCAGC	–	–
***Wolbachia***	–	–	–	–
wspF	F	TGGTCCAATAAGTGATGAAGAAACTAGCTA	(Jeyaprakash and Hoy 2000)	53 °C
wspR	R	AAAATTAAACGCTACTCCAGCTTCTGCAC	–	–

The CO1 alignment consisted of 64 successfully sequenced *S.globosum* specimens and 1239 bp of CO1, which included the original “barcode region” amplified by the Folmer primers (first 648 bp) and the CO1 terminal region obtained with an additional primer set (remaining 591 bp) (Table 1). All CO1 sequences were without double peaks and overall, of good quality. We added eight *S.globosum* specimens (from Germany, Austria, Switzerland and Bulgaria) with the original barcode from BOLD (Ratnasingham and Hebert 2007, 2013) to this alignment (Suppl. material 2), which resulted in a final CO1 alignment of 72 *S.globosum* sequences. The outgroup was built of eight sequences also obtained from BOLD, which corresponded to seven different species from the family of crab spiders (Thomisidae) and from two closely related families (Philodromidae and Sparassidae). For the nuclear ITS2 gene, we obtained 379 bp from 64 *S.globosum* specimens and added two Genbank (Benson et al. 2007) sequences of the outgroup taxon *Cymbacha* (Thomisidae) (Suppl. material 2). All specimens (N=64) were tested for *Wolbachia* by trying to amplify specific gene of *Wolbachia* DNA in spider samples using the primer pair wspF/wspR (for conditions see Table 1). Positive amplifications were visualised on an electrophoresis gel.

### ﻿Molecular data analysis

All sequences were prepared for analysis with MEGA7 (Kumar et al. 2016). Both CO1 and ITS2 alignments were constructed with the MUSCLE package as implemented in MEGA7 under default parameters. As a quality control, we checked CO1 sequences for stop codons and gaps, none of which were found.

Bayesian phylogenies were reconstructed in MrBayes version 3.2.6 (Ronquist et al. 2012). We used PartitionFinder 2.1.1 (Lanfear et al. 2012) to infer the partitions and whether the substitution models should include among character rate variation and proportion of invariable sites (settings: search = greedy, branchlengths = linked, model_selection = AICc). While the results suggest that all three codon positions should be analysed as different partitions, we combined first and second positions because the second positions showed almost no variation at all and thus should not be used to infer substitution rates. No such partitioning was applied for ITS2 since it is not a protein coding gene. Finally, a mixed substitution model was used to sample over the complete model space (Huelsenbeck 2004), while the among character rate variation and proportion of invariable sites were modelled according to the results of PartitionFinder. Markov Chain Monte Carlo (MCMC) sampling was conducted with one cold and three heated chains for 20 million generations, sampling every 1000^th^.The starting tree was not specified. For summary statistics, the first 50% of samples were discarded as a burn-in. We assumed convergence of the analyses when the average standard deviation of split frequency (ASDSF) was below 0.01, effective sample size was above 200 and likelihood graphs indicated stabilisation. For maximum likelihood (ML) estimation, we used RAxML (Stamatakis 2014). We performed 1000 bootstrap replicates under the GTRCAT model with a rapid search for bootstrap support and an exhaustive search for the ML tree. For the input files of MrBayes and RAxML and for the ITS2 consensus tree, see Suppl. material 4, 5. Trees were edited in FigTree Version 1.4.2 (Rambaut 2019) and additionally with Affinity publisher.

To quantitatively assess potentially overlooked species within *S.globosum*, the Bayesian Poisson tree processes (bPTP) method was applied, as a single marker method for species delimitation (Zhang et al. 2013). For the analysis, the Bayesian majority rule consensus tree with outgroup was used to delimit species. The analysis was ran on the bPTP server (https://species.h-its.org/ptp/) for 100'000 MCMC iterations with a burn in of 0.1. The number of generations was enough to reach convergence. For ITS2 and CO1, a haplotype network with PopArt (Leigh and Bryant 2015) implemented TCS network (Clement et al. 2002) was made to trace potentially different haplotypes. Because the PopArt software excludes all the sites with ambiguous nucleotides and gaps, for the haplotype reconstruction of ITS2, we excluded the sequences that introduced ambiguities at all but one parsimony informative sites. The exempted site contained too many ambiguities across alignment, thus we preferred that the analysis excludes this site to loosing substantial number of sequences and geographical information.

### ﻿Morphological analysis

In total, 61 specimens were successfully used for the morphological analysis. For each of the 34 adult females, four or five photographs were taken showing the dorsal and ventral views of the habitus, the ventral view of the opisthosoma, epigyne and vulva. For each of the 28 adult males, six pictures were made, showing the dorsal and ventral view of the habitus and the ventral, prolateral, dorsal, and retrolateral view of palps. Habitus and palp pictures were stacked from multi-focus records under a LEICA M205 C stereomicroscope with the corresponding IMS client software package. Body size measurements were also performed with the IMS client software. The pictures of epigynes and vulvae were taken on the digital microscope Keyence VHX -500F. If necessary, pictures were edited (i.e., corrected for brightness and contrast) with paint.NET (Brewster 2017) and Adobe Photoshop CS4. Additionally, all epigynes, vulvae and palps in ventral and retrolateral view were drawn in 122 sketches. Palps were fixed in glycerole gelatine and drawings were made under the Leica MZ 16 stereomicroscope with a 1.6× Planapo objective. The vulvae were embedded in Hoyer’s medium and drawn with a Zeiss Axioplan 2 compound microscope. Damaged (N = 1) and juvenile (N = 3) specimens were excluded from the morphological analysis. The following traits were examined: continuity of the black pattern on the dorsal side of opisthosoma; red, yellow or white colouration of the female opisthosoma; variation of the white stripe on the ventral side of opisthosoma; number of teeth on the prolateral and retrolateral claw of leg I; number of spines on metatarsus I; colouration pattern of male femora III and IV; cymbium of male palps, size and overall shape of the retrolateral tibial apophysis (RTA), the base of the RTA (BRTA), tibial apophysis (TA) and the embolus tip; on removed epigyne, shape of copulatory duct, receptaculum seminis, fertilisation duct and vulva hood. A morphological data matrix with all mentioned data was made, see Suppl. material 6. The four most promising out of these 12 measurements (colour, teeth of the prolateral claw on leg one, percentage of the white colour in male femora VI and if the vulval hood extends over the entrance) were then plotted on the CO1 Bayesian consensus tree, to see the evolution of morphology patterns according to CO1 clades (Fig. 3). The version R 4.1.1 (Rcore Team 2021) and the packages “ape” (Paradis and Schliep 2019) and “ctv” (Zeileis 2005) were used.

### ﻿Multivariate ratio analysis

For the morphometric data analysis, we used multivariate ratio analysis (MRA) by Baur and Leuenberger (2011). MRA comprises a commonly applied set of tools for explorative data analysis. It is especially useful for addressing questions in systematics and evolutionary biology (Baur et al. 2014; Petrović et al. 2017; Huber and Schnitter 2020; Selz et al. 2020; Nagy et al. 2021; Schmidt et al. 2021). Here we calculated a shape PCA and plotted the resulting shape PCs against isometric size (i.e., the geometric mean of all variables). We furthermore computed the PCA ratio spectrum for finding the most important character ratios with respect to a particular shape PC.

Reliability of variables used in the morphometric analysis followed the procedure described by Bailey and Byrnes (1990). However, calculation was done following Wolak et al. (2012) and by using their implementation in the R package ICC. Note, that we apply here reliability (R) in relation to measurement error (M) as: R = 1-M.

**Figure 2.**
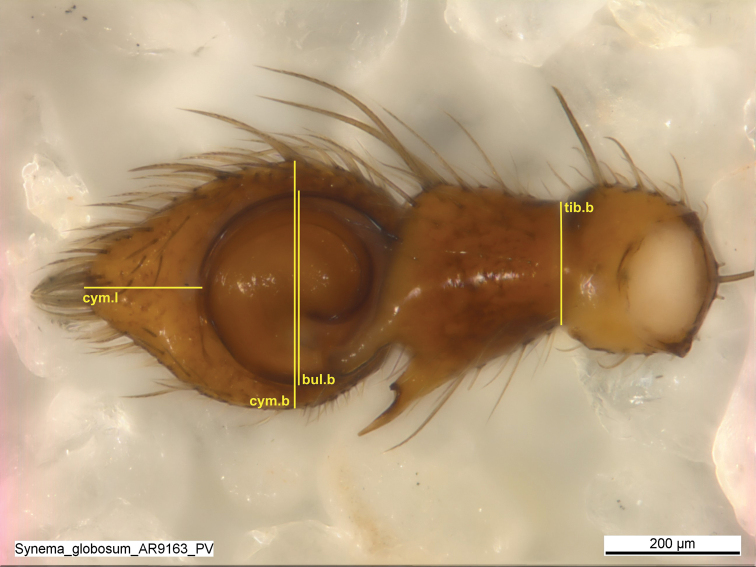
Measurements of the palp: Cym.l is the distance of the anterior margin to the tip of the cymbium, cym.b is the maximum breadth of the cymbium, bul.b is the maximum breadth of the genial bulbus and tib.b is the breadth of the tibia at the base of the patella joint.

The morphometric data set contained a few missing values. There were imputed with the help of the R package MICE (Buuren and Groothuis-Oudshoorn 2011) by using the default settings of the function mice(). Four measurements of palp were taken (see Table 2 and Fig. 2). Concerning the male palp, the images showed the palp from the ventral side. The structures were chosen because of the good defined start and endpoints. Difficulties to pose the palp in alcohol led to different positions in the pictures and hindered to take measurements of the bulb, RTA and TA. All four measurements were repeated 4×, so that we could perform the reliability analysis. One male specimen was excluded (AR10769). The measurements were done with IMAGEJ (Schneider et al. 2012). The morphometric analysis was performed with R Studio (RCore Team 2020) and the R-script from Baur and Leuenberger (2020). The results were visualised using package ggplot2 (Wickham 2016). Measurements on the female genitalia were not conducted because of the structural instability. Images of palps used for the morphometric analysis as well all R-scripts used for the MRA and reliability analysis are available on Zenodo (Urfer and Baur 2021).

**Table 2.** Measurements of the male palp.

Abbreviation	Character name	Definition
cym.l	Cymbium length	Distance of the anterior margin to the tip of the cymbium
cym.b	Cymbium breadth	widest breadth of the cymbium
bul.b	Bulb breadth	widest breadth of the genital bulbus
tib.b	Tibia breadth	breadth of the tibia base at the patella joint

**Figure 3.**
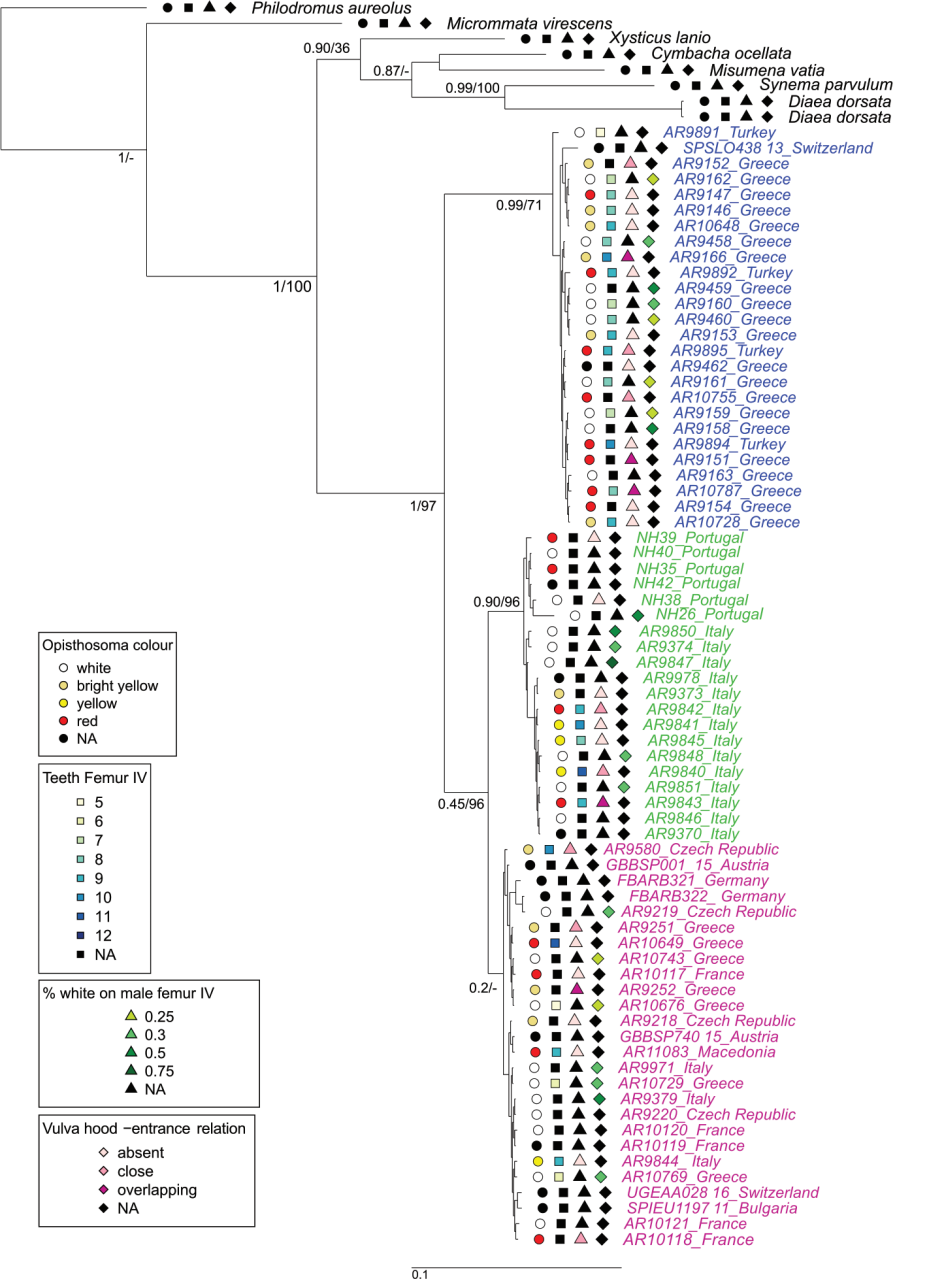
Bayesian majority rule consensus tree for CO1. The analysis included 72 individuals of *Synemaglobosum* and eight outgroup sequences. Node supports represent Bayesian posterior probabilities/ML bootstrap support based on 1,000 replicates; colours correspond to the three distinct clades. The specimen labels contain country information after the specimen number. Four different symbols before each specimen correspond to the states of four scored morphological traits; circles indicate the colour of the opisthosoma, squares the number of teeth on the prolateral claw of leg one, upside triangles the percentage of white colour starting at the base of leg IV in males, downside triangles the entrance state of the vulval hood; black filled symbols indicate a not applicable state (NA).

## ﻿Results

### ﻿Molecular analysis

The 1239 bp long CO1 alignment contained 113 polymorphic sites. The CO1 tree showed clear support for the monophyly of *S.globosum*. The Bayesian and ML analyses of CO1 both inferred three clades (Fig. 3). These clades were not geographically separated and occurred sometimes within the same sample location (Figs 1, 3). The mean uncorrected p-distance between clade one and clade two was approximately 6% and between clade one and clade three 5.5% (Table 3). We recovered the outgroup as expected, except for *S.parvulum* (Hentz, 1847) from America which grouped closer to *Diaeadorsata* (Fabricius, 1777) and rendered the genus *Synema* Sundevall 1833 paraphyletic. In the combined CO1 and ITS2 Bayesian analysis, we had poor convergence according to the ASDSF value (> 0.03). Therefore, we excluded the concatenated tree from our study.

**Table 3.** Mean uncorrected p-distances between and within the CO1 clades of *S.globosum*.

CO1 clades	Clade 1	Clade 2	Clade 3
Clade 1	0.002	–	–
Clade 2	0.061	0.004	–
Clade 3	0.053	0.029	0.004

For the CO1 tree with the outgroups, the bTPT analysis suggested between nine and 13 species. The best ML and Bayesian solution considered seven outgroup species and three highly supported (pp > 0.7) species within *Synemaglobosum*. The haplotype network of *S.globosum* showed a slight geographic pattern with two main haplotypes: one dominantly containing Greek specimens plus one specimen from Switzerland and the second from Turkey with another haplotype containing Italian and Portuguese specimens (Fig. 5). The other haplotypes showed no clear geographical pattern and contained individuals from a few countries.

**Figure 4.**
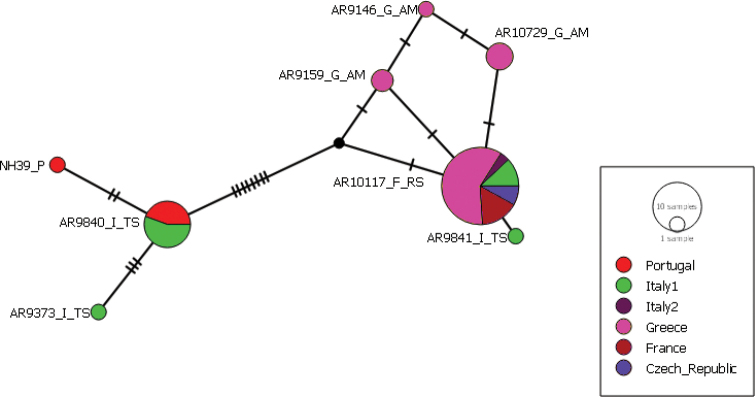
ITS2 haplotype network of *Synemaglobosum*. Nodes represent haplotypes with the size corresponding to the frequency of the haplotype. The short black lines represent mutations. The colours represent the countries of origin of sequences and have no relation with the CO1 clades.

In ITS2 16 out of 379 positions were variable according to the PopArt setting based on the reduced dataset; ten of these 16 were parsimony informative. The network showed two dominant haplotypes with no clear geographic pattern (see haplotype network, Fig. 4). According to SeqStat four nucleotide indels were found. There were three gaps of one nucleotide and one indel of two nucleotids. One of the two nucleotide indels was heterozygous in an individual from Italy. The uncorrected pairwise distance in ITS2, calculated with MEGA7 ranged from 0.00 to 0.05 with mean of 0.01. In the endosymbiont screening all individuals were tested for *Wolbachia* but in only one individual, we had a positive amplification on the electrophoresis gel. Since there was a proper positive control, the positive specimen was not sent for sequencing.

**Figure 5.**
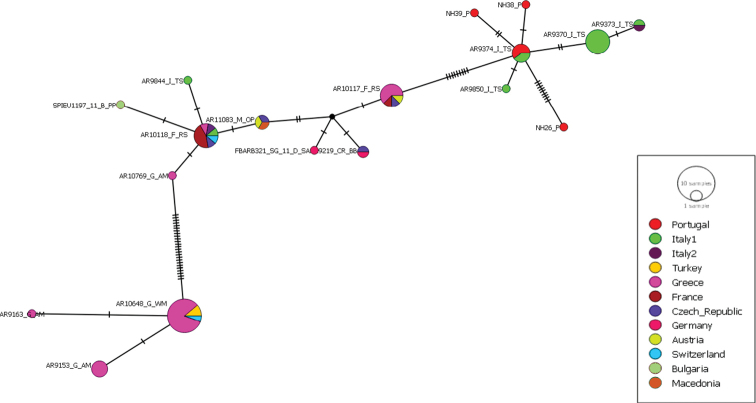
CO1 haplotype network of *Synemaglobosum*. Nodes represent different haplotypes with the size corresponding to the frequency of the haplotype. The short black lines represent mutations. The colours represent the countries of the origin of the sequences.

### ﻿Morphological analysis

**Figure 6.**
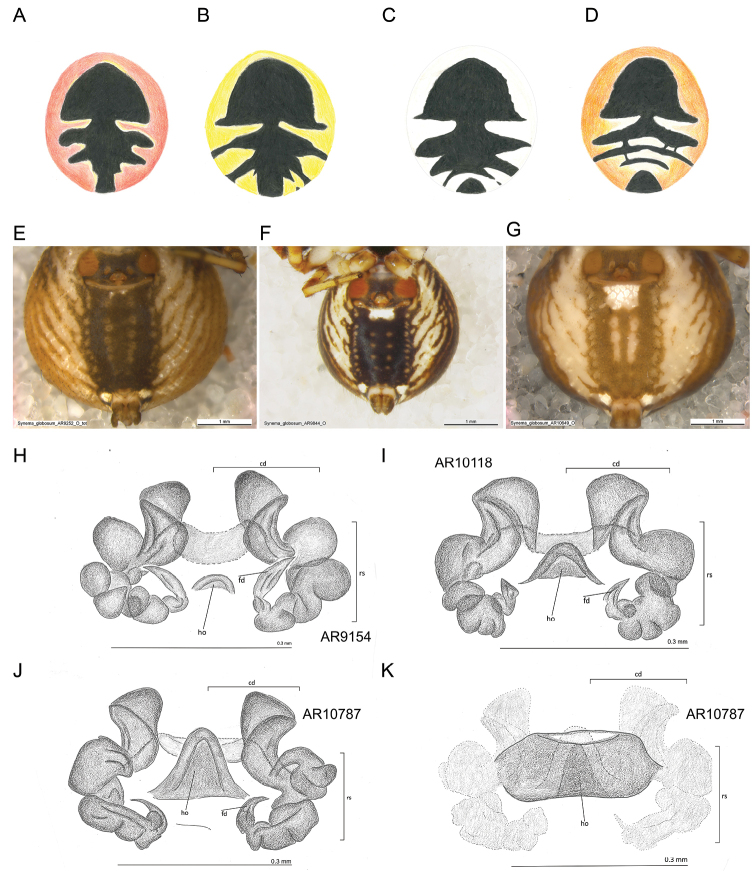
Variation in morphology in the female. **A–D** opisthosoma, dorsal view, colour and black pattern variation **E–G** white stripe on the ventral side of opisthosoma **E** Greece, Peloponnese **F** Italy, Tuscany **G** Greece, west Macedonia **H–J** variation in the vulva **H** Greece, Marathonas **I** France, Savoy **J** Greece, west Macedonia **K** epigyne of the specimen from **J** with very deep hood. Abbreviations: cd = copulatory duct, rs = receptaculum seminis, fd = fertilisation duct, ho = hood.

**Figure 7.**
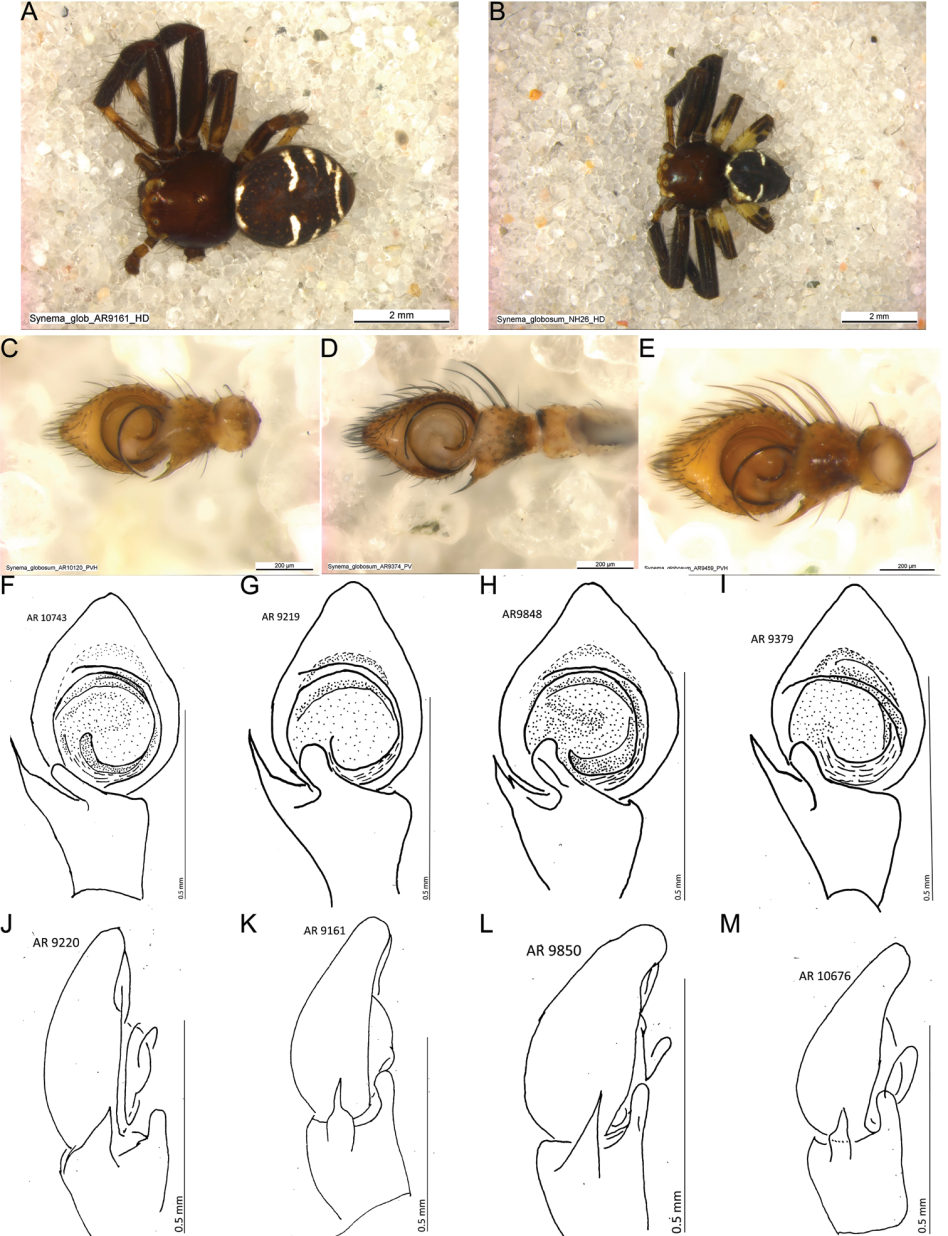
Variation in morphology in the male I. **A, B** habitus males with different colour pattern on femora III and IV **A** Greece, Marathonas **B** Portugal **C–E** Different sizes of palp in ventral view **C** France, Savoy **D** Italy, Siena **E** Greece, Marathonas **F–I** palp, ventral view, the variation of the retrolateral tibial apophysis and the tibial apophysis **F** Greece, West Macedonia **G** Czech Republic, Brno **H, I** Italy, Tuscany **J–M** retrolateral view of the palp, variation in the retrolateral tibial apophysis **J** Czech Republic, Brno **K** Greece, Attiki **L** Italy, Siena **M** Greece, west Macedonia.

**Figure 8.**
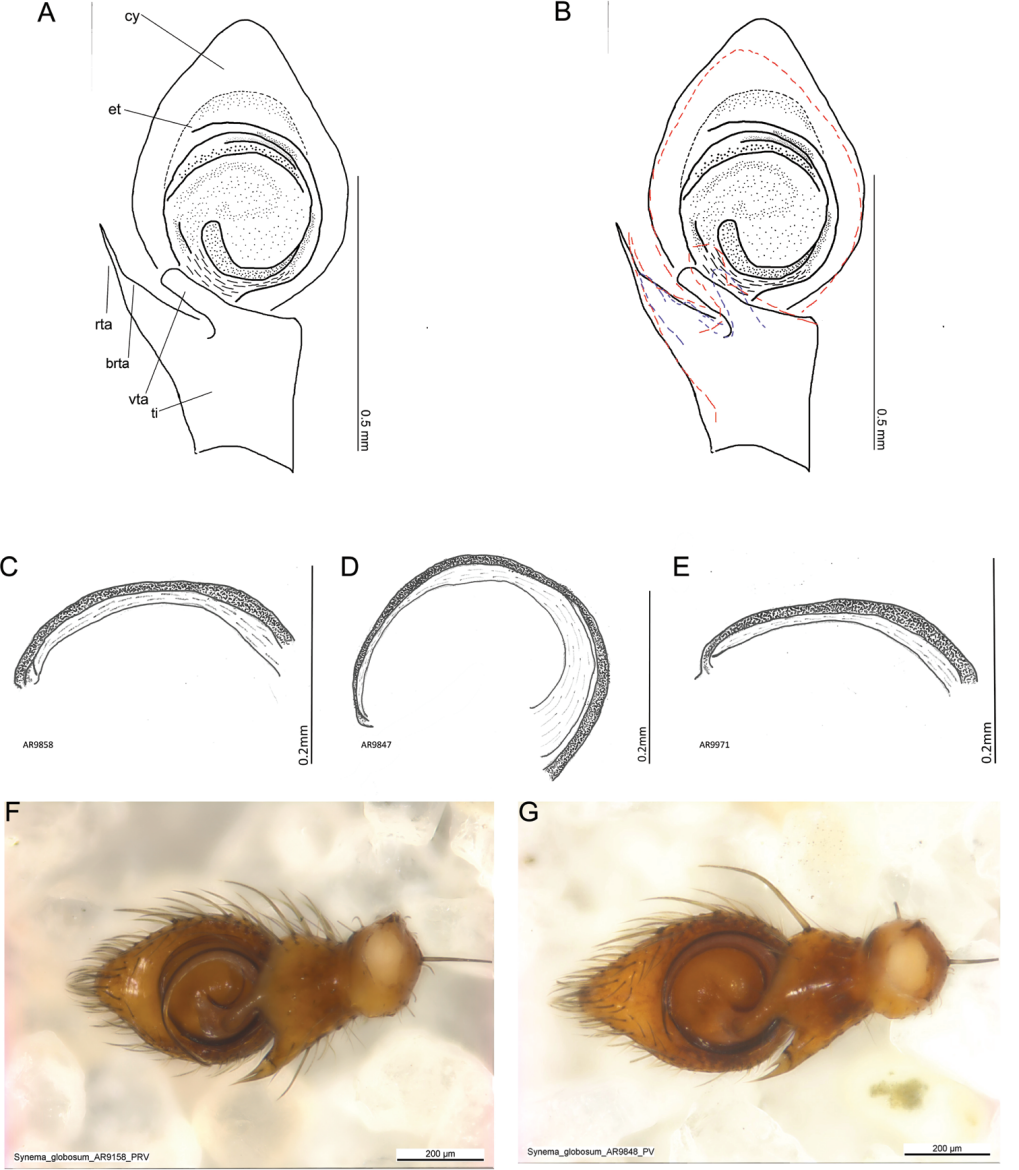
Variation in morphology in the male II. **A** palp with all variable structures **B** outlines of the palps from two additional males that showadditional variation **C–E** tips of embolus **C** Italy, Siena **D, E** Italy Toskana **F–G** two out of three individuals where the rta shows a second tip **F** Greece, Marathonas **G** Italy, Tuscany. Abbreviations: cy cymbium, et embolus tip, rta retrolateral tibial apophysis, brta base of the retrolateral tibial apophysis, ti tibia, vta ventral tibial apophysis

The morphology of *S.globosum* showed extensive variation in both sexes in almost every structure that was examined (Figs 6–8), but none of this variation showed any correspondence to the CO1 clades geography. The number of teeth on the claws of leg I varied from five to eleven and the difference between the prolateral and the retrolateral claw was small (± 1 tooth). The number of spines on metatarsus I and metatarsus II varied from three to four.

**Female morphology.** The average body size in females was 5.5 mm (3.48 mm to 7.83 mm). The female opisthosoma had a red, yellow or white ground colour. The black colour pattern on the dorsal side of the opisthosoma was unique to each specimen, in some being continuous and in others interrupted in various ways (Fig. 6A–D). Ventrally on the opisthosoma, there was often a white stripe behind the epigyne, but this could also be entirely absent (Fig. 6E–G). Neither the colour nor the colour patterns covaried with the CO1 clades. The vulvae showed large variation (Fig. 6H–K). The copulatory duct contained cashew nut-shaped structures with various degrees of bending. The position of the *receptaculum seminis* varied, as does the position of the fertilisation duct. The two structures were closely connected and may influenced each other’s position. The vulva hood showed large depth variation ranging from almost absent to the point where the hood overlapped the copulatory opening (Fig. 6H–K). No correspondence between these structures and the CO1 clades was found.

**Male morphology.** The average body size in males was 3.8 mm (2.94–4.56 mm). In contrast to females, they showed only black and white opisthosoma colour, with a much higher amount of black than white, sometimes small white coloured females could be confused with males. The black pattern on the opisthosoma was not always continuous (Fig. 7A, B). The ventral white stripe was short (usually) or absent (only in few individuals). Notable variation occurred in femora III and IV, which were either brownish or black or they were basally bright and then darker towards the apical part (Fig. 7A, B).

One trait used for identifying *S.globosum* males was the tibia of the palp, which was longer than wide (Levy 1985). This was confirmed in every examined male. However, the palps differed strongly in size (Fig. 7C–E), the height and shape of the cymbium, and the shape and size of the RTA. The TA showed less variation in shape and more in its position (Figs 7F–M, 8A, B). The embolar duct twisted 1.5× and ended distally with the embolus tip (Fig. 8A; Levy 1985). The tip of the embolus was without a thickened end (Fig. 8C–E). The tip of the RTA was needle-like. In three specimens from Italy and Greece, the base of the RTA is extended, seemingly forming a second, shorter tip (Fig. 8F, G). None of the examined structures matched the CO1 clades or showed geographical clustering.

### ﻿Morphometric data analysis

Body measurements were first inspected concerning reliability (R). The latter was generally high to very high, with only a single character showing R = 89%. All other character had R > 95%. A table with confidence intervals together with a bar plot were available in the Zenodo repository (Urfer and Baur 2021).

**Figure 9.**
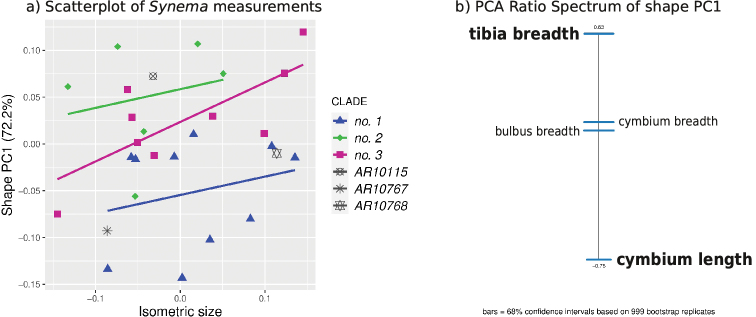
**A** Shape PC1 plotted against isometric size of 28 males. Colours correspond to the CO1 clades. **B** PCA Ratio Spectrum for shape PC1. The three specimens with grey symbols could not be included in the molecular analysis and therefore could not be attributed to a clade. Regression lines follow a least-squares model.

Only shape PC1 was significant, which explained 72.2% of the variation. It showed only very slight differentiation among the clades, which overlapped strongly (Fig. 9A). The PCA ratio spectrum revealed that shape variation was mostly related to the ratio of tibia breadth to cymbium length (tib.b/cym.l), and the importance of the other ratios must be considered negligible.

## ﻿Discussion

The analysis of 64 *S.globosum* specimens showed an astonishingly high variation in morphological traits as well as in the mitochondrial gene CO1 and, to a much less extent, in the nuclear gene ITS2. While this could indicate overlooked species within *S.globosum*, the lack of a clear relationship between the groups delimited by molecular data and morphological variation or geographical distribution is not in favour of the cryptic species hypothesis or of an ongoing speciation process. The results rather suggest a single, highly variable species. However, for a final solution of this problem, more molecular data are needed, for example obtained with whole genome or ddRAD sequencing together with testing for mating barriers in *S.globosum*.

### ﻿CO1 gene with three distinct clades

Barcoding can be used to accurately distinguish higher taxonomic groups, e.g., genus and family level in spiders (Čandek and Kuntner 2015; Coddington et al. 2016; Kennedy et al. 2020) and is nowadays commonly used as a helpful tool to support red list assessments and species inventories (Blagoev et al. 2013; Astrin et al. 2016; Crespo et al. 2018; Gregorič et al. 2020). Finding such deep CO1 clades within one species was thus unexpected. Deep CO1 mitochondrial divergence without speciation has been reported for several organisms such as the common redstart *Phoenicurusphoenicurus* (Hogner et al. 2012), gall wasps (Nicholls et al. 2012) and in the butterfly genus *Heliconius* (Muñoz et al. 2011). In arachnids, similar findings concern groups with a low dispersal ability such as mygalomorphs (Arnedo and Ferrández 2007) or species with major geographical barriers in their distribution area (Chamberland et al. 2020). Since there is evidence that *S.globosum* is able to balloon (Blandenier and Fürst 1998) and it is quite common in Europe, the low dispersal ability should not be the reason of the deep CO1 divergence.

The bTPT analysis suggested three species that correspond to the three CO1 clades identified in the phylogenetic analyses. It is a helpful tool for single-locus species delimitation, however Blair and Bryson (2017) suggested to treat the results with caution and to analyse at least a second nuclear marker, as single markers (and especially uniparentally inherited ones) might provide an incomplete picture.

### ﻿Discordant patterns of nuclear and mitochondrial phylogenies in *S.globosum*

The ITS2 phylogeny of 64 *S.globosum* specimens did not reflect the CO1 clade pattern. ITS2 is a nuclear rRNA marker that is assumed to mutate via concerted evolution (Elder and Turner 1995). The number of ITS2 copies is very high in the genome, and the individual copies usually show no variation among each other because DNA repair mechanisms homogenise their sequences within the genome (Zimmer et al. 1980; Elder and Turner 1995; Álvarez 2003). Ortiz et al. (2021) found the pattern of over-splitting CO1 when testing ddRAD sequencing in an ant-eating *Zodarion* species. The species was assumed to consists of two cryptic lineages based on ecological traits. They found over-splitting in number of species when only the CO1 barcode gap was analysed. This gap was not supported by the variation in ITS2.

On the other hand, it is a plausible assumption, that CO1 barcodes can reflect cryptic speciation and ITS2 has low substitution rate which is too low to catch the interspecific distances. However, this should always be verified with a larger molecular study.

Haplotype analyses based on mtDNA markers can indicate ancient geographic structures (Nicholls et al. 2012). Thus, we could suggest that the slight geographic pattern identified in the CO1 haplotype network may reflect isolated populations in glacial refugia. However, ITS2-based networks do not support such an interpretation. A constraint to testing this hypothesis is the biased sampling towards eastern Europe, leading to an incomplete representation of west European haplotypes. To assess population structuring more appropriately in *S.globosum*, we suggest additional taxon sampling, especially of populations on the Iberian Peninsula, and application of more suitable molecular markers for population-level genetic analyses.

Introgression from a related species by past hybridisation events is a second scenario that could explain deep CO1 clades (Galtier et al. 2009). However, it does not explain the morphological variation to the extent we found it. Alternatively, despeciation after secondary contact might explain the CO1 clades as well as the high genetic and morphologic variation in *S.globosum.* Finding traces of despeciation could thus explain the patterns observed here. However, de-speciating lineages are very difficult to trace, even with large amounts of genetic data and are beyond the scope of our study (Taylor et al. 2006; Kearns et al. 2018).

Infection with endosymbiotic bacteria that may alter the mitochondrial structure of species (Hurst and Jiggins 2005; Goodacre et al. 2006) is an additional possible scenario. It offers a possibility for hybridisation and introduction of haplotypes into a species, in this way distorting single locus CO1 barcoding (Narita et al. 2006; Klopfstein et al. 2016). Since we found only a single infection, the probability of this scenario is also unlikely. But it should be kept in mind that an endosymbiotic infection has occurred very early and no traces of it are left today.

### ﻿High morphological variation in *S.globosum*

The morphometric analysis of the male palp showed only a very slight differentiation among clades in the first shape PC, but in general the clades overlapped strongly. We found high and continuous variation in the colour pattern and the shape of the epigyne, vulva and palp in all examined populations of *S.globosum* (Fig. 3). Unusually high morphological variation is reflected by the number of described subspecies (Dahl 1907; Franganillo 1913, 1926b; World Spider Catalog 2021), which are nowadays considered unfounded and were probably described due to insufficient knowledge of the extent of intraspecific variation in this species. This shows that the assessment of intraspecific variation is underestimated and should be included more in taxonomic studies.

The main characters used to delimit species of spiders are found in the genitalia (e.g., Huber 2004). However, intraspecific genitalic variation in spiders is largely understudied and rarely accounted for in identification keys (e.g., Levy 1985; Mcheidze 1997; Utochkin 1960b). Genitalic variation, especially in male copulatory organs, is predicted by the theory of cryptic female choice, which states that parts of the male’s genital bulb are supposed to serve as copulatory courtship devices enabling the female to evaluate the male’s quality during mating (e.g., Eberhard 1996; Kuntner et al. 2009). Cryptic female choice could therefore play a role in shaping the morphological diversity of *S.globosum* palps. However, for a deeper understanding of these problems, further studies on the mating behaviour and mechanic coupling of the copulatory organs are necessary.

In our study we had a biased sample size mostly towards easter Europe. In this region, the species *Synemacaucasicum* Utochkin, 1960, occurs regularly. The separation of *S.caucasicum* from *S.globosum* is based on the colour pattern on the ventral side of the opisthosoma, where *S.caucasicum* shows five brighter marks. The palp of *S.caucasicum* looks almost identical to that of *S.globosum*, and the epigyne structure lies within the variation that we recorded in *S.globosum*. *S.caucasicum* is endemic to Georgia and Azerbaijan (Khasayeva and Huseynov 2019; Utochkin 1960b) and it remains unclear if it just represents a local morph of *S.globosum*, as there are only insufficient drawings, sketches and descriptions of *S.caucasicum* available. Because a precise examination of the *S.caucasicum* taxonomy was beyond the scope of this study, this problem should be addressed in further investigations.

## ﻿Conclusions

Based on a large set of specimens of *S.globosum* from a wide geographical range, we found three deep clades in the CO1 gene tree and large variation but no resolution in the ITS2 gene tree. We also found remarkable intraspecific morphological variation in sexual organs and in other characters that are commonly used for species delimitation. However, this variation does not show any geographical pattern or correspondence with the CO1 clades. In order to better understand the high morphological variability in *S.globosum*, we suggest looking at a larger molecular dataset, such as multilocus phylogeny based on restriction-site associated DNA markers (Peterson et al. 2012) or whole genome sequences conducted on a broader geographic range, which can capture processes at or below the current species level of *S.globosum*.

## Supplementary materials

Supplementary material 1 Coordinates of the collected specimens Data type occurences https://binary.pensoft.net/file/624781This dataset is made available under the Open Database License (http://opendatacommons.org/licenses/odbl/1.0/). The Open Database License (ODbL) is a license agreement intended to allow users to freely share, modify, and use this Dataset while maintaining this same freedom for others, provided that the original source and author(s) are credited.Karin Urfer , Tamara Spasojevic , Seraina Klopfstein , Hannes Baur , Liana Lasut , Christian Kropf

Supplementary material 2 Genbank and Bold numbers of the *Synemaglobosum* specimens that were obtained from these databases Data type genomic https://binary.pensoft.net/file/624782This dataset is made available under the Open Database License (http://opendatacommons.org/licenses/odbl/1.0/). The Open Database License (ODbL) is a license agreement intended to allow users to freely share, modify, and use this Dataset while maintaining this same freedom for others, provided that the original source and author(s) are credited.Karin Urfer , Tamara Spasojevic , Seraina Klopfstein , Hannes Baur , Liana Lasut , Christian Kropf

Supplementary material 3 GenBank accession numbers Data type Genomic https://binary.pensoft.net/file/624783This dataset is made available under the Open Database License (http://opendatacommons.org/licenses/odbl/1.0/). The Open Database License (ODbL) is a license agreement intended to allow users to freely share, modify, and use this Dataset while maintaining this same freedom for others, provided that the original source and author(s) are credited.Karin Urfer , Tamara Spasojevic , Seraina Klopfstein , Hannes Baur , Liana Lasut , Christian Kropf

Supplementary material 4 Input file Mr Bayes and RAxML Data type Phylogenetic Explanation note The exact input scripts that we used to run RAxML and MrBayes analysis. https://binary.pensoft.net/file/624784This dataset is made available under the Open Database License (http://opendatacommons.org/licenses/odbl/1.0/). The Open Database License (ODbL) is a license agreement intended to allow users to freely share, modify, and use this Dataset while maintaining this same freedom for others, provided that the original source and author(s) are credited.Karin Urfer , Tamara Spasojevic , Seraina Klopfstein , Hannes Baur , Liana Lasut , Christian Kropf

Supplementary material 5 Bayesian majority rule consensus tree of ITS2 Data type Image Explanation note The analysis included 64 *S.globosum* specimens and two outgroup sequences. Node supports represent Bayesian posterior probabilities/ML bootstrap support based on 1,000 replicates. Colours correspond to the CO1 clades, black indicates the absence of a CO1 sequence. The last two letters are the country code according to ISO 3166. https://binary.pensoft.net/file/624785This dataset is made available under the Open Database License (http://opendatacommons.org/licenses/odbl/1.0/). The Open Database License (ODbL) is a license agreement intended to allow users to freely share, modify, and use this Dataset while maintaining this same freedom for others, provided that the original source and author(s) are credited.Karin Urfer , Tamara Spasojevic , Seraina Klopfstein , Hannes Baur , Liana Lasut , Christian Kropf

Supplementary material 6 Morphomatrix of the examined *Synemaglobosum* individuals Data type Morphological https://binary.pensoft.net/file/624786This dataset is made available under the Open Database License (http://opendatacommons.org/licenses/odbl/1.0/). The Open Database License (ODbL) is a license agreement intended to allow users to freely share, modify, and use this Dataset while maintaining this same freedom for others, provided that the original source and author(s) are credited.Karin Urfer , Tamara Spasojevic , Seraina Klopfstein , Hannes Baur , Liana Lasut , Christian Kropf
