# Supplementary figures and images for: ﻿Incongruent molecular and morphological variation in the crab spider Synemaglobosum (Araneae, Thomisidae) in Europe
Source: Zookeys. 2021 Dec 17;1078:107–34. doi: 10.3897/zookeys.1078.64116 (PMC8709837; doi:10.3897/zookeys.1078.64116)

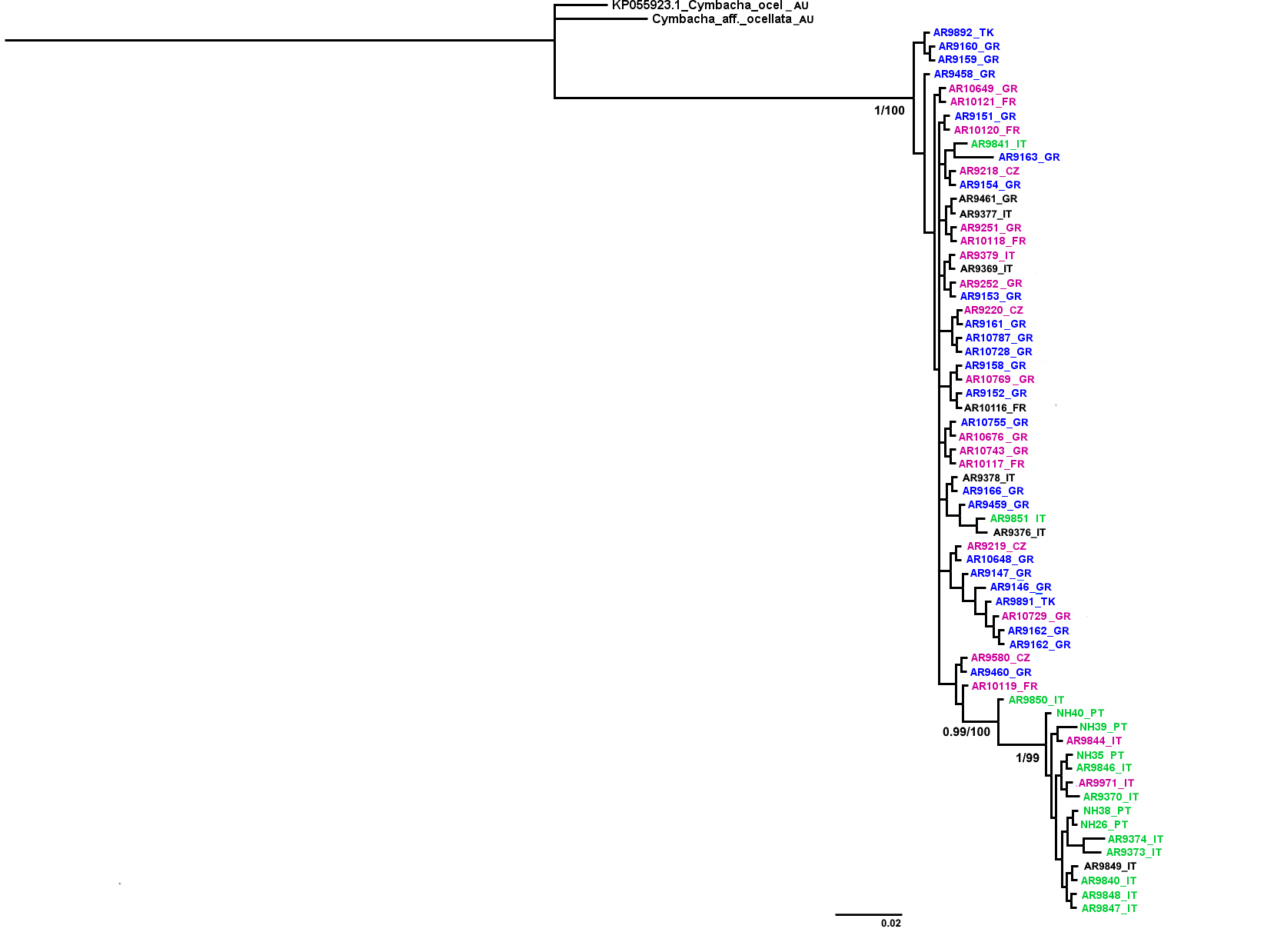

Supplement: Supplementary material 5 — Bayesian majority rule consensus tree of ITS2 [file zookeys-1078-107-s005.png]
